# Glutathione Peroxidase from *Talaromyces marneffei* Interacts with Host Cytoskeletal Proteins: Insights from Yeast Two-Hybrid and Molecular Dynamics Simulations

**DOI:** 10.3390/ijms27104259

**Published:** 2026-05-11

**Authors:** Tanaporn Wangsanut, Yin Htet Htet Aung, Yin Htet Htet Oo, Narin Lawan, Monsicha Pongpom

**Affiliations:** 1 Department of Microbiology, Faculty of Medicine, Chiang Mai University, Chiang Mai 50200, Thailand; 2 Department of Chemistry, Faculty of Science, Chiang Mai University, Chiang Mai 50200, Thailand

**Keywords:** glutathione peroxidase, talaromycosis, MD simulation, protein–protein interaction, moonlighting proteins, FKBP15 (FKBP133 or WAFL), host cytoskeleton remodeling

## Abstract

*Talaromyces marneffei* is a dimorphic fungal pathogen that can subvert host immune defenses; however, the molecular mechanisms underlying its interactions with host cells remain incompletely understood. Glutathione peroxidase from *T. marneffei* (TmGpx1) has previously been identified as an antigenic protein that elicits antibody responses in patients with talaromycosis. To elucidate the contribution of TmGpx1 during human–fungal pathogen interaction, the yeast two-hybrid system was performed using TmGpx1 as bait to screen a cDNA library derived from non-induced human macrophage cells. Sixteen candidate host protein partners were identified, with Gene Ontology analysis revealing their predominant association with cytoskeletal and extracellular exosome components. To examine the atomic-level structural interface and dynamic behavior of protein–protein interactions, we employed molecular dynamics (MD) simulations to investigate the interaction between TmGpx1 and FKBP15, a human protein involved in early endosomal regulation and associated with both microtubule and actin filaments. Per-residue decomposition analysis using gmx_MMPBSA identified LEU124 of TmGpx1 and ARG616 of FKBP15 as critical residues mediating the protein–protein interaction. Notably, the key residues of TmGpx1 are located toward the N-terminus and are mapped outside of the catalytic active site, suggesting that the interaction of TmGpx1 with host cytoskeletal components may occur independently of its enzymatic antioxidant activity. Overall, our findings provide novel insights into the repertoire of host cytoskeletal and membrane trafficking proteins that may be targeted for remodeling during *T. marneffei* infection. Elucidation of these molecular interactions advances our understanding of host–pathogen dynamics and opens new avenues for the development of targeted diagnostics and therapeutic strategies against talaromycosis.

## 1. Introduction

Talaromycosis, also known as penicilliosis, is the invasive mycosis caused by the dimorphic fungus *Talaromyces marneffei* [1]. New talaromycosis cases are reported annually, with a significantly high mortality rate approaching one-third of the infected individuals [2]. The clinical severity in patients can vary widely, ranging from localized to systemic infection, depending on the individual’s underlying conditions and immune status. Infections confined to a single site are rare and typically present with only localized symptoms, without evidence of dissemination. In contrast, immunosuppressed patients, whether or not they are HIV-positive, are more likely to develop systemic infections. Common manifestations include fever, weight loss, fatigue, lymphadenopathy, hepatosplenomegaly, respiratory, and gastrointestinal abnormalities [3]. *T. marneffei* grows as a saprophytic mold at 25 °C but undergoes a thermally regulated transition to a pathogenic yeast form at 37 °C [4]. After inhalation of asexual conidia and their attachment to host tissues, phagocytic cells represent the first line of defense against *T. marneffei* infection. Conidia are phagocytosed by resident alveolar macrophages as well as polymorphonuclear neutrophils (PMNs). Within macrophages, conidia rapidly undergo a morphogenic transition to the yeast form and replicate intracellularly by binary fission [5,6].

As an intracellular fungal pathogen, *T. marneffei* employs multiple mechanisms to survive and proliferate within macrophages. First, thermal dimorphism is essential for dissemination and is linked to the virulence [7]. Inside macrophages, *T. marneffei* switches from the mold to the yeast form and disseminates via a “Trojan horse” mechanism that protects the fungus from neutrophil-mediated killing and promotes systemic spread. Second, heat shock proteins such as HSP90 and HSP70 regulate morphogenesis and support survival under environmental stress, including temperature, pH, and osmotic stress [8]. Third, *T. marneffei* expresses key antioxidant enzymes to defend against oxidative stress. For instance, genes encoding catalase peroxidase (CpeA), superoxide dismutase (SodA), aspartyl protease (PepA and Pop) are upregulated during macrophage infection [9,10,11]. Collectively, these mechanisms enable *T. marneffei* to exploit macrophages as a protected niche for replication and dissemination, primarily through the reticuloendothelial system [12].

Glutathione peroxidase is an important antioxidant that catalyzes the reduction of hydroxyperoxides, using glutathione as a cofactor. There are two states of glutathione in the cells: reduced glutathione (GSH) and oxidized glutathione disulfide (GSSG). In fact, glutathione peroxidase catalyzes the following reaction: 2GSH + H_2_O_2_ → GSSG + 2H_2_O. In our previous study, the glutathione peroxidase gene from *T. marneffei* (TmGpx1) was isolated as one of the antigenic proteins that can stimulate host antibody production in immunocompromised patients with talaromycoses [13]. The cDNA library was constructed from the pathogenic yeast phase of *T. marneffei* and screened for antigenic protein-encoding genes using a pooled antibody derived from *T. marneffei*-infected patients [10,13]. Thus, TmGpx1 contributes to the immunological response during fungal infection. However, it is unknown how TmGpx1 functions during host–pathogen interaction.

The dynamics of the host cytoskeleton and membrane systems are central players in host–pathogen interactions. As the cytoskeleton governs the formation of membrane extensions, adhesion complexes, and junction integrity, intracellular bacteria are known to subvert the host cytoskeleton to facilitate invasion and spread [14,15]. In addition, many bacterial pathogens manipulate host membrane trafficking to accommodate their substantial size, supplying necessary membranes for invasion, intracellular growth, and eventual dissemination throughout host tissues [16]. Similarly, viral pathogens extensively remodel host cell membranes and cytoskeletal networks to establish specialized organelle-like structures known as viral factories, which concentrate the cellular machinery required for viral genome replication [17]. These viral factories are often encapsulated by cage-like formations of actin, microtubules, or intermediate filaments, underscoring the critical role of the cytoskeleton in pathogen replication and survival. Altogether, emerging research areas have highlighted the sophisticated strategies employed by bacterial and viral pathogens to reroute host membrane trafficking and reorganize host cytoskeleton, revealing the cytoskeleton and endomembrane system as central targets in the cellular response to microbial infection.

While extensive research has demonstrated that cytoskeletal dynamics and membrane trafficking undergo extensive remodeling during bacterial and viral invasion, little is known about these processes during fungal infection. In this study, we aimed to determine whether the glutathione peroxidase TmGpx1 participates in host–pathogen interactions beyond its antioxidant role. Specifically, we set out to (i) identify human host proteins that interact with TmGpx1 using a yeast two-hybrid screen, (ii) prioritize cytoskeletal and membrane-trafficking-related partners, and (iii) use molecular dynamics (MD) simulations to map the putative interaction interface and key contact residues on TmGpx1. By integrating experimental interaction screening with computational interface analysis, we provide evidence supporting a potential moonlighting function of TmGpx1 during infection.

## 2. Results

### 2.1. The Human Host Macrophage Interactome by TmGpx1

As the TmGpx1 is detected in extracellular vesicles [18,19], we hypothesized that TmGpx1 could localize outside the fungal cells and directly interact with human host proteins. To identify the host proteins that could potentially interact with TmGpx1 during *T. marneffei* infection, the yeast two-hybrid assay was conducted. As *T. marneffei* can infect multiple mammalian host cell types, the non-induced human macrophage offers the best representation of the human proteome encountered by *T. marneffei* during initial host infection. Accordingly, the full-length of the TmGpx1 gene was used as bait, and the cDNA library of a non-induced macrophage cell line was used as prey. The assay shows that the lexA-bait is neither toxic nor autoactivating. There are 258 positive clones obtained from the screen, and 16 interactants were identified with very high and high confidence scores (Table 1). Gene Ontology analysis revealed that the identified proteins are significantly associated with the microtubule plus-end, the cortical cytoskeleton, the cell cortex, and extracellular vesicle components, including exosomes (Table 2). This suggests that the protein set likely functions in the maintenance of cellular structure, intracellular trafficking, and intercellular communication via extracellular vesicles.

**Figure 1:**
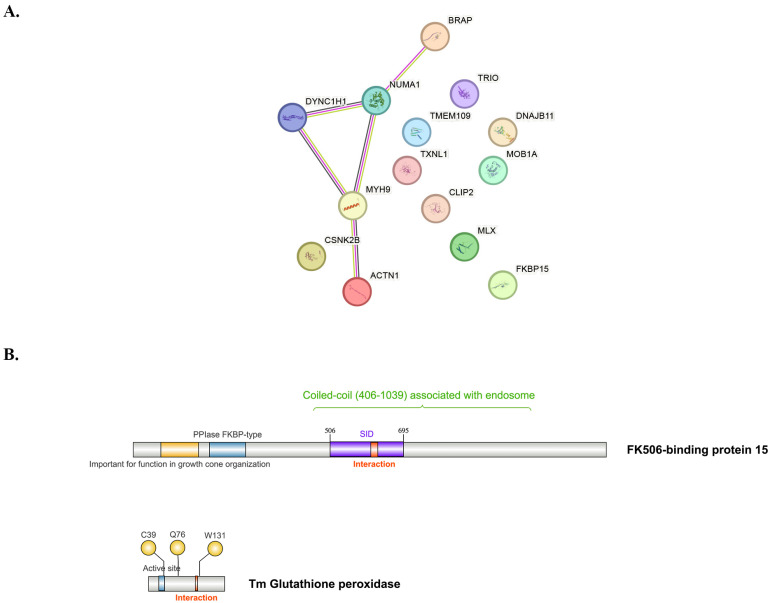
**TmGpx1 Interactome Recovered from the Yeast Two-Hybrid Experiment.** (**A**) STRING protein–protein interaction (PPI) network generated from the human macrophage proteins recovered as putative TmGpx1 interactors in the yeast two-hybrid screen (TmGpx1 from *T. marneffei* as bait; non-induced human macrophage cDNA library as prey). Nodes represent host proteins identified in the yeast two-hybrid assay. Edges represent host–host functional associations from STRING (i.e., known or predicted relationships among the host proteins) and do not correspond to bait–prey interactions measured directly by yeast two-hybrid experiment. Network statistics: 14 nodes, 5 edges (expected edges = 1), average node degree = 0.714, average local clustering coefficient = 0.262, PPI enrichment *p*-value = 0.00223. (**B**) Putative functional domains of TmGpx1 and FKBP15 were annotated based on protein alignment and MD simulation analyses. The schematic illustrates both conserved regions and newly identified key residues. Amino acid positions are indicated along the schematic, and newly mapped interaction regions are highlighted in red boxes. SID = Selected Identified Domain, identified by yeast two-hybrid experiment.

To further characterize the functional relationships among the proteins identified by the yeast two-hybrid experiment, a protein–protein interaction (PPI) network was constructed using the STRING database (Figure 1A). Importantly, the connections shown in this network represent host–host functional associations curated or predicted by STRING and do not represent bait–prey interactions measured directly by the yeast two-hybrid assay. The network contained 14 nodes and 5 edges (expected edges = 1) and showed significant PPI enrichment (*p* = 0.00223), indicating that the identified host candidates are more functionally connected than expected by chance. The resulting network revealed prominent interactions among key cytoskeletal and cell division-related proteins, including ACTN1 (Alpha-actinin-1), MYH9 (Myosin-9), DYNC1H1 (Cytoplasmic dynein 1 heavy chain 1), NUMA1 (Nuclear mitotic apparatus protein 1), and BRAP (BRCA1-associated protein). Strong associations were observed between ACTN1 and MYH9, which are critical components of the actin cytoskeletal network, mediating F-actin cross-linking and cellular contractility, respectively. Furthermore, both DYNC1H1 and NUMA1 are central to microtubule-based processes, specifically mitotic spindle formation and function, essential for chromosome segregation during cell division. The interaction between NUMA1 and DYNC1H1 underscores their coordinated role in spindle pole organization and mitotic progression. BRAP, although less directly structural, is implicated in the regulation of signaling pathways that may modulate cytoskeletal dynamics and nuclear–cytoplasmic transport. Collectively, the STRING analysis supports that the yeast two-hybrid-derived host candidates cluster into a coherent functional module related to cytoskeletal organization, microtubule dynamics, and cell division.

In summary, the yeast two-hybrid screen, together with downstream bioinformatics analyses, identified host interaction candidates for TmGpx1 that are enriched in cytoskeletal and membrane-trafficking functions. This enrichment supports the hypothesis that TmGpx1 may engage host structural and trafficking pathways during infection. These findings also provided the rationale for selecting representative host partners for structural modeling and MD simulations to map the putative binding interface and key contact residues.

### 2.2. MD Simulation of TmGpx1 and Host Protein FKBP15

#### 2.2.1. Selection of a Host Protein That Interacts with TmGpx1

Based on the results presented in Table 2 and Figure 1, we speculated that *T. marneffei* utilizes TmGpx1 to manipulate host cytoskeletal dynamics and membrane reorganization, thereby promoting its survival during macrophage infection. Although FKBP15 (also known as FKBP133 and WAFL) was not initially identified within the core list of either canonical cytoskeletal proteins or membrane-associated exosomes from our gene ontology enrichment, it was prioritized for further investigation based on two main criteria. First, the yeast two-hybrid protein–protein interaction screening revealed a high-confidence interaction score (PBS A, Table 1) for FKBP15 with the bait protein. Second, a literature review indicates that FKBP15 is a membrane-binding protein that is critically involved in endosomal trafficking and the regulation of early endocytic transport [20,21]. As *T. marneffei* survives in the phagosomal compartment, interference with endosome and phagosome fusion is important. Indeed, FKBP15 binds to WIP (WASP-interacting protein) and actin proteins to move early endosomes, then changes function as endosomes reach microtubules, acting as a scaffold to assemble factors involved in vesicle movement [20]. Expression studies indicate that FKBP15 is upregulated during monocyte differentiation into macrophages, which exhibit highly dynamic membrane activity and robust environmental sampling [20]. Furthermore, the presence of FKBP15 on phagosomes containing invasive bacteria, such as *Salmonella*, *Yersinia*, and *Shigella*, suggests a direct role in phagocytosis [22]. Together, these findings position FKBP15 as a key regulator of vesicular trafficking and cytoskeletal coordination, particularly within highly active immune cells such as macrophages.

Given the potential bridging role in cytoskeletal coordination and the strong interaction observed in our yeast two-hybrid screen, we selected FKBP15 for subsequent MD simulations to explore its structural and dynamic properties in complex with TmGpx1.

#### 2.2.2. Functional Domain Analysis and MD Simulations

FKBP15 contains an N-terminal PPIase FKBP-type domain (including an FK506-binding and peptidyl-prolyl cis-trans isomerase domain) located at residues 197–290 (Figure 1B (top)). It also has a large coiled-coil domain spanning residues 522–789, 818–878, and 925–951. The central coiled-coil region (residues 406–1039) mediates association with early endosomes [20]. Our yeast two-hybrid results indicate that residues 506–695 of FKBP15 interact with TmGpx1 (Figure 1B (top)), suggesting that TmGpx1 may disrupt the interaction between FKBP15 and early endosomes, as well as with the associated cytoskeleton.

TmGpx1 is predicted to function as an antioxidant enzyme, with a conserved active site located at the N-terminal residues 27–42 (Figure 1B (bottom)). This site is believed to enable the neutralization of reactive oxygen species (ROS) by catalyzing the reduction of H_2_O_2_ to H_2_O. The predicted active site contains a conserved selenium–cysteine (U) or cysteine (C) residue, together with glutamine (Q) and tryptophan (W), forming the characteristic “catalytic triad” of the glutathione peroxidase family [23]. However, the specific regions of TmGpx1 that interact with host proteins were not identified from our yeast two-hybrid screen, as the full-length TmGpx1 was used as bait. To address this, MD simulations were performed to predict and refine the regions of TmGpx1 that potentially mediate interactions with potential host partners.

**Figure 2:**
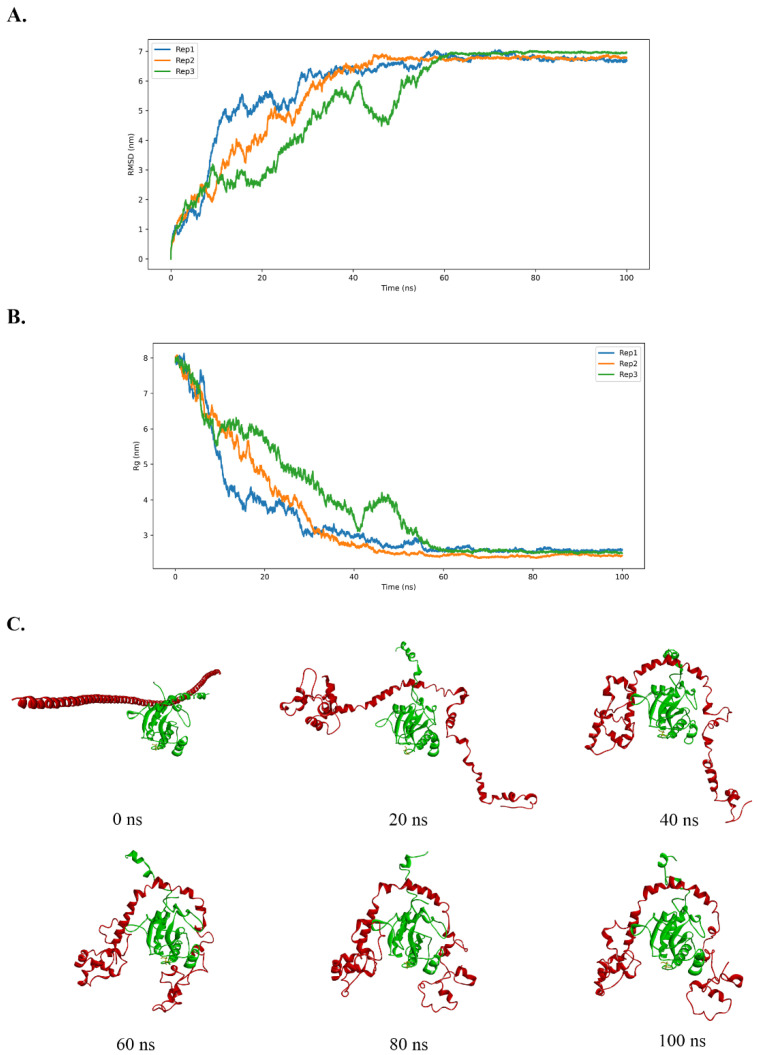
**MD simulation of TmGpx1 from *T. marneffei* and human FKBP15 was performed for 100 ns.** (**A**) Root mean square deviation (RMSD) of the protein atoms was calculated to assess the stability of the TmGpx1–FKBP15 complex over time. RMSD is reported with respect to the starting structure; the plateau regions (25–40 ns onward) were used qualitatively to indicate convergence. (**B**) Radius of gyration (Rg) of the protein complex as a function of time, used to monitor overall compactness during the simulation. (**C**) Representative structural snapshots of the TmGpx1–FKBP15 complex (replicate 1) were extracted at 0, 25, 50, 75, and 100 ns to visualize conformational changes over the course of the simulation. MD simulations were conducted in three replicates. Red: FKBP15 and Green: TmGpx1.

The initial structure of the protein complex was predicted by AlphaFold3 using FKBP15 residues 506–779, encompassing the interaction region and coiled-coil domain, together with the full-length TmGpx1 protein. To assess the reliability of the modeled starting conformations, we benchmarked the predicted TmGpx1 monomer structure against experimentally determined homologs using the RCSB PDB structure-alignment tool. The AlphaFold3-predicted TmGpx1 superimposed well with human GPx4 (PDB: 2GS3, chain A) (RMSD 1.11 Å, TM-score 0.90, 38% sequence identity; 160 aligned residues) and with the fungal homolog yeast Hyr1 (PDB: 3CMI, chain A) (RMSD 1.67 Å, TM-score 0.92, 54% sequence identity; 140 aligned residues) (Figure S1 and Table S1). These comparisons support that the modeled TmGpx1 fold used to initiate MD simulations is consistent with experimentally observed GPx structures. Next, MD simulations of the FKBP15-TmGpx1 complex were conducted for 100 ns to evaluate structural stability, using the AMBER99SB force field. To determine whether a 100 ns production run was sufficient, we extended one independent simulation (replica 1) to 200 ns and assessed convergence using the time evolution of the 3D structure together with root-mean-square deviation (RMSD) and radius of gyration (Rg) profiles (Figure S2A,B). These metrics indicate that the system reaches a stable, converged regime by 100 ns, supporting the use of 100 ns trajectories for subsequent analyses. As shown in Figure 2, the RMSD (calculated relative to the initial structure) increased during the early relaxation phase and then plateaus after 25–40 ns in all three replicates, with subsequent fluctuations around a stable mean, consistent with convergence to a stable conformational regime of the complex. For the remainder of the simulation, RMSD values fluctuated within a relatively narrow range, suggesting maintenance of the complex’s overall structural integrity without large-scale conformational changes.

The Rg was calculated to monitor the overall compactness of the protein complex during the 100 ns MD simulation (Figure 2B). The Rg profile exhibits a pronounced decrease from 8 nm at the beginning of the trajectory (0 ns) to 2 nm by approximately 60 ns, after which it remains stable at 2 nm until the end of the simulation. This behavior indicates that the large RMSD relative to the initial structure reflects a major conformational rearrangement toward a more compact state rather than progressive unfolding/expansion of the complex (Figure 2A,C). The stabilization of Rg after 60 ns further suggests that the system reaches a relatively stable and compact conformation over the latter part of the trajectory.

#### 2.2.3. Protein–Protein Binding Free Energy

To evaluate the robustness of the predicted interaction, MM-GBSA calculations were performed on three independent MD simulations (Table S2). All replicates yielded negative total binding free energies, indicating energetically favorable complex formation. The calculated ΔG binding values were −120.24 ± 14.18 kcal/mol (replicate 1), −55.39 ± 8.91 kcal/mol (replicate 2), and −87.12 ± 8.09 kcal/mol (replicate 3). The average binding free energy across replicates was −87.58 ± 32.43 kcal/mol (Table 3). The binding free energy analysis revealed that binding was strongly driven by gas-phase interactions, particularly van der Waals and electrostatic contributions. The mean van der Waals contribution across replicates was −182.28 ± 41.29 kcal/mol, showing relatively consistent stabilization. Electrostatic interactions were substantial (−744.01 ± 300.03 kcal/mol) but showed greater variability across simulations. These favorable electrostatic interactions were largely counterbalanced by polar solvation effects (ΔG polar = 864.63 ± 304.76 kcal/mol), indicating compensation between intermolecular electrostatic interactions and the energetic costs of solvent. In contrast, the nonpolar solvation term consistently contributed favorably to binding (ΔG nonpolar = −25.93 ± 5.93 kcal/mol). Although variability was observed in the electrostatic and polar solvation components, the van der Waals and nonpolar contributions were relatively consistent across replicates, suggesting that hydrophobic interactions play a dominant role in stabilizing the TmGpx1–FKBP15 complex. The convergence of the MM-GBSA binding free energy estimate was further assessed by extending one independent simulation (replica 1) to 200 ns and evaluating the binding energy over the entire trajectory (Figure S2C). The mean binding free energy averaged over the full 200 ns trajectory was −112.42 kcal/mol. Consistent with the structural stability indicated by the RMSD and radius of gyration profiles after 100 ns (Figure S2A,B), the binding-energy profile did not show a systematic drift in the later portion of the simulation, supporting that the reported binding free energy is representative for the timescale studied.

**Figure 3:**
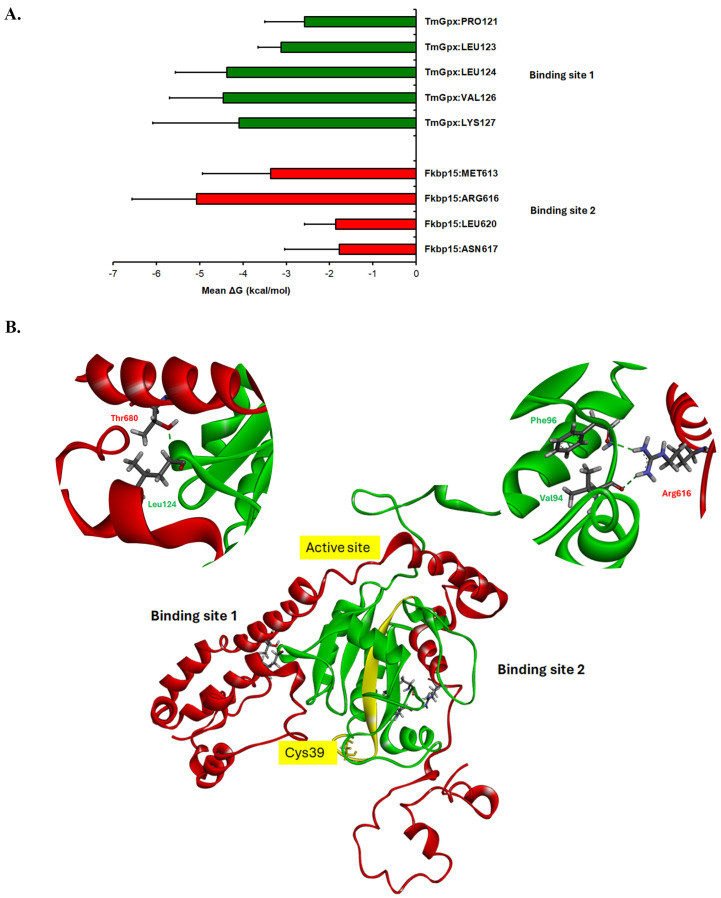
**Per-residue binding free energy decomposition and structural identification of interaction sites in the TmGpx1–FKBP15 complex.** (**A**) Total decomposition contributions (TDC) of selected interfacial residues calculated using the MM-GBSA approach over the 90–100 ns trajectory. Negative ΔG values indicate favorable contributions to binding. Error bars represent standard deviations. (**B**) Putative interaction interface identified by combining structural proximity analysis and per-residue energy decomposition. Residues within ≤12 Å were defined as interface residues. Binding site 1 highlighting LEU124 of TmGpx1, identified as a primary intermolecular contact residue based on a ≤6.2 Å distance cutoff. Binding site 2 highlighting ARG616 of FKBP15, also identified as a primary contact residue within the ≤6.2 Å cutoff. Red: FKBP15 and Green: TmGpx1.

To identify key residues contributing to complex stabilization, per-residue energy decomposition analysis was performed using the MM-GBSA method over the 90–100 ns trajectory. The analysis revealed several residues with consistently favorable binding energy contributions (ΔG < −2 kcal/mol), indicating stable involvement at the interaction interface (Figure 3A). Notably, per-residue interface energy decomposition performed independently for each of the three MD replicates consistently identified TmGpx1 LEU124 and FKBP15 ARG616 as the dominant contributors to the binding interface across all trajectories. On the TmGpx1 side (binding site 1), LEU124 exhibited the strongest and most consistent stabilizing contribution, with total energy values ranging approximately from −3 to −7 kcal/mol across the analyzed frames (Figure 3B). This contribution was predominantly driven by van der Waals interactions, suggesting tight interfacial packing. Additional residues, including LEU123, VAL126, and PRO121, also showed stable negative energy contributions, largely dominated by nonpolar interactions. On the FKBP15 side (binding site 2), MET613 and LEU620 displayed favorable contributions primarily arising from van der Waals interactions (Figure 3B). ARG616 showed substantial electrostatic interactions; however, these were partially offset by polar solvation penalties, resulting in a moderate net stabilization effect. A similar electrostatic–solvation compensation was observed for LYS127. In contrast, ARG630 exhibited minimal net contribution despite large electrostatic components, suggesting limited participation in stable interfacial contacts. Notably, structural analysis indicated that LEU124 of TmGpx1 forms an intermolecular hydrogen bond with THR680 of FKBP15 (Figure 3B), while ARG616 of FKBP15 forms hydrogen bonds with VAL94 and PHE96 (Figure 3B). Overall, the energy decomposition analysis indicates that the complex is stabilized primarily by hydrophobic packing and shape complementarity, with van der Waals interactions representing the dominant energetic contribution. Electrostatic interactions appear to modulate interfacial orientation but are not the main determinants of binding stability.

#### 2.2.4. Force-Field Sensitivity Analysis

To evaluate force-field sensitivity, we compared AMBER99SB and CHARMM36m simulations [24]. The protein RMSD profile and representative 3D structures suggest that the AMBER99SB trajectory underwent larger changes in the relative orientation/arrangement of the protein–protein complex (Figure 2A,C). In contrast, the CHARMM36m simulation maintained a more similar overall complex geometry (Figure S3). These observations suggested force-field-dependent differences in the simulated complex behavior.

## 3. Discussion

While the role of the cytoskeleton and membrane remodeling in host interactions with human fungal pathogens remains largely elusive, recent research has highlighted the plant cytoskeleton as a central player in plant immunity and a key target of fungal pathogen virulence factors [25,26,27]. In plant models, cytoskeletal remodeling in response to pathogen attack is critical for coordinating various defense mechanisms, including immune receptor trafficking, membrane organization, organelle aggregation, and the transport of defense compounds [27]. Importantly, changes in the cytoskeleton also modulate plant immune responses by influencing salicylic acid signaling, reactive oxygen species (ROS) generation, and the expression of defense-related genes [27]. During infection by fungal pathogens or oomycetes, plants often exhibit increased actin filament density at sites of pathogen entry, reflecting active cytoskeletal reorganization [28,29,30]. For example, actin filaments in the epidermal cells of *Arabidopsis* leaves undergo marked rearrangement following infection with the barley powdery mildew fungus *Blumeria graminis* f. sp. *hordei* (Bgh) [31]. Similarly, *Magnaporthe grisea* alters actin dynamics by increasing actin density within plant cells [32]. Collectively, these findings underscore the pivotal and dynamic role of the cytoskeleton in plant–fungal pathogen interactions.

Our study highlights the potential for the human cytoskeleton and membrane to serve as primary targets for fungal virulence factors. By performing a yeast two-hybrid assay in human macrophage cells, we suggest that cytoskeletal and membrane remodeling may occupy a crucial intersection between host immune responses and fungal adaptation. An intriguing question is whether host immune cells actively remodel their cytoskeleton and membranes to counter fungal invasion, as observed in plant–fungal pathogen interactions, or whether fungal pathogens manipulate these host structures within immune cells to promote their own survival, a strategy commonly employed by intracellular bacteria and viruses. Addressing this question will be of significant interest for future investigations, as it promises to advance our understanding of host–pathogen interactions in intracellular pathogens at the cellular level. While many findings clearly demonstrate that cytoskeleton and membrane remodeling are common responses to microbial infection [26,27,33], the specific patterns and mechanisms of rearrangement can vary depending on the type of pathogen involved. To date, no studies have examined the interaction between *T. marneffei* and host cytoskeletal networks or membrane structures. This makes it particularly interesting to investigate how TmGpx1, a glutathione peroxidase, interacts with these host components. Notably, while pathogen effectors are typically secreted to hijack host cellular machinery [26,33], TmGpx1 is not classified as an effector in the conventional sense. Therefore, further research is warranted to elucidate the unique mechanisms by which TmGpx1 may influence or interact with the host cytoskeleton dynamics and membrane trafficking during infection.

Our per-residue energy decomposition analysis identified the key interaction region of TmGpx1 with FKBP15 at the C-terminus, distinct from the predicted active site at the N-terminus. This suggests that the role of TmGpx1 in binding FKBP15 may be functionally separate from its putative enzymatic antioxidant activity. However, we note that the interacting residues are clustered near TRP131, a residue that forms part of the catalytic triad. Therefore, it is possible that the host–pathogen interaction may not be entirely independent of the enzyme’s catalytic function. The insights gained from our MD simulations provide a valuable foundation for future functional studies, such as site-directed mutagenesis [34,35]. LEU124 of TmGpx1 was identified as a key interaction residue based on per-residue MM-GBSA decomposition. It consistently exhibited strong negative energy contributions, predominantly driven by van der Waals interactions, indicating a central role in hydrophobic packing at the interface. Structural analysis further showed that LEU124 is among the closest intermolecular contact residues (≤6 Å) and forms a hydrogen bond with THR680 of FKBP15. Together, these findings suggest that LEU124 acts as a stabilizing hotspot, contributing to complex formation primarily through tight packing and local interfacial complementarity. Experimental validation through site-directed mutagenesis (e.g., L124A substitution) would help confirm its functional importance in mediating the TmGpx1–FKBP15 interaction.

Most importantly, this is the first study to report the second function of glutathione peroxidase in addition to its canonical antioxidant function. Our findings suggested that TmGpx1 could serve as a moonlighting protein, performing multiple, distinct functions beyond its primary, well-characterized role [36,37]. The diversity of moonlighting activities often depends on cellular context, localization, interaction partners, or specific cellular conditions. In humans, for example, glutathione peroxidase 4 (GPx4) is recognized as a moonlighting protein, exhibiting both enzymatic and non-enzymatic roles [38,39]. While GPx4 acts as a scavenger of lipophilic hydroperoxides, it can also be converted to an enzymatically inactive structural form within the cell [39]. In fact, research in the model yeast *Saccharomyces cerevisiae* demonstrated that, for one-third of the enzymes examined, deletion phenotypes could be complemented by versions of the protein lacking catalytic activity [37], suggesting that these proteins have important functions beyond their enzymatic roles. Numerous moonlighting proteins have been linked to the virulence of both bacterial and fungal pathogens [40]. Most of these are highly conserved housekeeping proteins, typically involved in chaperone activity, stress response, or metabolic processes. For instance, glyceraldehyde-3-phosphate dehydrogenase (GAPDH), a known moonlighting protein, has been shown in *T. marneffei* to act as an adhesin that facilitates cell attachment to the lung epithelium, in addition to its classical role in glycolysis [41]. The pathogenic potential of such moonlighting proteins is often linked to their secretion or relocalization into the extracellular environment [37]. Extracellular vesicles (EVs) may facilitate the relocalization of moonlighting proteins by transporting them to new cellular environments or even to other cells. Through EV-mediated transfer, a moonlighting protein can be exposed to different partners or compartments, allowing it to adopt alternative functions that would not occur in its original location. Recent proteomic analysis has identified glutathione peroxidase among 394 proteins present in EVs derived from *T. marneffei*, supporting the hypothesis that TmGpx1 may moonlight during infection [18]. Likewise, GPx1/3/4 isoforms in humans and animals have been detected in EVs isolated from various tissues, including stem cells, cardiac tissue, adipose tissue, and plasma [42]. Altogether, these findings support the notion that TmGpx1 could function not only as an antioxidant enzyme but also as a structural or signaling component that interacts with host proteins during fungal infection. The mechanism enabling this functional switch may involve changes in cellular localization, particularly through EV-mediated transport.

Our study has some limitations. First, binding free energy was estimated using the MM-GBSA end-point approach based on equilibrated MD trajectories, which provides a practical and computationally efficient, but approximate, measure of binding affinity. We note that more rigorous free-energy methods, such as umbrella sampling, can be used to compute a potential of mean force (PMF) along a defined reaction coordinate and may yield more accurate absolute binding free energies. However, such PMF calculations require careful selection of the reaction coordinate, window spacing, and extensive convergence testing, and were therefore beyond the scope of the present work. Future studies will consider umbrella sampling/PMF calculations to further refine the free-energy characterization. Second, despite using independent replicas (mean MM-GBSA binding free energy across three replicas: −87.58 ± 32.43 kcal/mol) and verifying time stability of RMSD/Rg and MM-GBSA profiles, we note that a strict proof of ergodicity for protein–protein association is rarely achievable within finite MD times. Therefore, our estimates should be interpreted as converged within the conformational basin(s) sampled over 100–200 ns, based on the observed stability of structural (RMSD/Rg) and MM-GBSA time-series profiles, while enhanced sampling (e.g., REMD/REST) or longer trajectories could further broaden conformational exploration and narrow uncertainty. Third, despite the experimental evidence for interaction from yeast two-hybrid screening, the binding interface and key contact residues proposed in this work are derived from structure prediction and MD simulations and therefore require experimental validation. In particular, the absence of site-directed mutagenesis to test predicted interface residues is a major limitation of the present study. Future work will prioritize mutational analysis of recurrent TmGpx1 interface residues identified across multiple host partners, followed by biochemical and/or cellular interaction assays to confirm their contributions to binding. In addition, because the simulations were performed using a fragment of FKBP15 rather than the full-length protein, the modeled interaction interface may not fully capture potential contributions from other FKBP15 regions or the full-length conformational context. This FKBP15 fragment is enriched in charged residues and is expected to adopt predominantly helical/coiled-coil-like conformations, a class of sequences that often lacks close experimental structural homologs and may populate multiple conformational states. Therefore, the MD-derived interaction features should be interpreted within the sampled conformational ensemble rather than as a single definitive binding mode.

## 4. Materials and Methods

### 4.1. Yeast Two-Hybrid Assay

ULTImate yeast two-hybrid assay was conducted by Hybrigenics Service (Paris, France). The coding sequences for *T. marneffei* TmGpx1 (PMAA_007230, Hyr1) strain ATCC18224 was synthesized and cloned into the Y2H vector (Hybrigenics, France). The LexA-TmGpx1 bait was screened for potential interacting partners by mating with yeast containing a randomly primed non-induced human macrophage cDNA library (prey). Positive clones were isolated, and the corresponding prey fragments were PCR-amplified, sequenced, and identified using the NCBI GenBank database and BLAST (version 2.17.0) analysis. To assess the reliability of each protein–protein interaction, a predicted biological score (PBS) was assigned to each partner candidate, ranging from the highest probability of specificity (A score) to the lowest (E score). Graphical representations of each interacting partner (Domsight) were analyzed (Supplemental Data S1), revealing minimal interacting domains identified in the screen, together with known functional domains or structural motifs.

### 4.2. Bioinformatics Analysis

Candidate interacting partners of the TmGpx1 protein were subjected to gene ontology (GO) enrichment analysis, using the PANTHER (Protein ANalysis THrough Evolutionary Relationships) Classification System (https://geneontology.org/ and https://pantherdb.org, accessed on 31 January 2026). The resulting protein–protein interaction network was further analyzed using the STRING database (https://string-db.org/, accessed on 31 January 2026) to visualize and assess connectivity among the identified partners. Visualization of protein functional domains and key residues was performed using the IBS 2.0 tool (https://ibs.renlab.org, accessed on 31 January 2026).

### 4.3. Protein Modeling

To predict protein complex structure, protein sequences for each protein were retrieved from NCBI and used as input (Supplemental Data S2). The 3D structural model of the protein–protein complex was computationally generated using https://alphafoldserver.com/, accessed on 24 March 2025, powered by the Alphafold3 model [43]. For each prediction, five models (model 0–4) were obtained. The top-ranked predicted models (models 0–3) were evaluated, and model 1 was selected as the starting structure for subsequent MD simulations based on visual inspection of the exported structure file (.cif), prioritizing a physically reasonable interface geometry. The AlphaFold3 confidence metrics for models 0–3 can be accessed at: https://github.com/N-Lawan/Y2H_gpx1_fkbp15.git, accessed on 30 April 2026. The corresponding ipTM scores were 0.17 (model 0), 0.14 (model 1), 0.14 (model 2), and 0.16 (model 3). In addition, pLDDT (per-atom confidence estimate) and PAE (predicted aligned error) scores were used to evaluate the model and guide selection for subsequent MD simulations [44].

### 4.4. Molecular Dynamics (MD) Simulation

MD simulations were performed using the GROMACS (GROningen Machine for Chemical Simulations) software package, version 2022.4, as previously described [45]. Briefly, the gmx pdb2gmx command was used to convert the initial molecular system prepared by AlphaFold3 (PDB file) into a GROMACS topology and format. The system topology and parameters were generated using the AMBER99SB force field. The protein–protein complex was positioned at the center of a cubic simulation box with a 1.0 nm distance from the box edge, and the box was filled with solvent (water) using the three-point TIP3P water model, prepared with the gmx editconf command. The system was neutralized by adding sodium and chloride ions to reach a physiological salt concentration of 0.15 M; ions were added using the gmx genion command after system preparation with gmx grompp. Energy minimization was executed with gmx mdrun -v -deffnm em, and was followed by equilibration in two stages: 100 ps NVT and 100 ps NPT (dt = 2 fs), using the gmx mdrun -v -deffnm nvt and gmx mdrun -v -deffnm npt commands, respectively. During equilibration, protein positional restraints were applied (-DPOSRES). Temperature was controlled using the V-rescale thermostat with two coupling groups (Protein and Water_Ion) at 310.15 K (tau_T = 0.1 ps). Pressure coupling was applied only in the NPT stage using the Parrinello–Rahman barostat (isotropic; 1 bar, tau_P = 2.0 ps; compressibility 4.5 × 10^−5^ bar^−1^). Equilibration was assessed by monitoring temperature and potential energy (NVT) and temperature, pressure/density, and potential energy (NPT) before initiating production MD. All simulations were performed at 310.15 K (37 °C) to represent human physiological temperature. The production run was performed for 100 ns under periodic boundary conditions (PBC) in all three spatial dimensions (pbc = xyz). Long-range electrostatic interactions were calculated using the particle mesh Ewald (PME) method (coulombtype = PME) with a real-space cutoff of 1.0 nm. Lennard-Jones (van der Waals) interactions were also computed using a 1.0 nm cutoff (rvdw = 1.0).

To analyze the dynamics of the protein complexes, trajectory files (.xtc files) were first concatenated using the gmx trjcat command, and all molecules were then centered within the box using gmx trjconv with the -pbc mol -center option. The structural stability of the protein complex was assessed using the gmx rms command, which calculates the root-mean-square deviation (RMSD). Three independent replicates were performed for each protein complex. The topology files, MD parameter files (.mdp), and the initial coordinate file (.gro) are publicly available. These files can be accessed at: https://github.com/N-Lawan/Y2H_gpx1_fkbp15.git, accessed on 30 April 2026.

### 4.5. Per-Residue Decomposition Analysis Using gmx_MMPBSA

To identify the contribution of individual amino acid residues to the binding free energy within the protein–protein complex, per-residue decomposition analysis was performed using the gmx_MMPBSA package [46]. The per-residue energy decomposition was carried out, using the script gmx_MMPBSA --create_input gb pb decomp (idecomp = 1), following the package’s user documentation. This approach allowed the separation of the total binding energy into contributions from each residue at the protein–protein interface. Results were analyzed and visualized using the gmx_MMPBSA_ana module, which generated tables and graphical representations of the energy contributions from each residue. Graphical representations were generated using Excel, and 3D models were visualized with BIOVIA Discovery Studio.

### 4.6. Computational Resources

MD simulations were performed on the CMU HPC ERAWAN system, comprising CPU nodes equipped with AMD EPYC 7742 (64 cores × 2 per node, 2.25 GHz) and AMD EPYC 9534 (64 cores × 2 per node, 2.45 GHz), and GPU nodes equipped with NVIDIA A100 80 GB and NVIDIA H100 80 GB GPUs. For the AMBER99SB simulations, each SLURM job requested 4 CPU cores per task and 1 A100 GPU, whereas for the CHARMM36m simulations, each SLURM job requested 64 CPU cores per task and 1 H100 GPU.

## 5. Perspective and Conclusions

This study employed a yeast two-hybrid approach using TmGpx1 as bait to identify sixteen candidate protein partners from a cDNA library of non-induced human macrophage cells. Subsequent bioinformatics analysis revealed that these interactors are predominantly associated with the cytoskeleton and extracellular exosomes, suggesting their potential involvement in cellular structural organization and vesicle-mediated processes. To further investigate these interactions at the atomic level, MD simulations were performed between TmGpx1 and FKBP15, allowing for the identification and mapping of key residues essential for protein–protein binding. Notably, investigating the molecular mechanisms underlying TmGpx1 interactions with host cytoskeletal and membrane structures could provide new perspectives for improving diagnostic development. For example, when TmGpx1 or its specific interaction partners, such as host cytoskeletal or membrane components, are found to be unique to *T. marneffei* infection or trigger distinctive host responses, these molecules and pathways can serve as highly specific targets for diagnostic development. Additionally, identifying unique cytoskeletal rearrangements or signaling pathways in host cells responding to *T. marneffei* may yield measurable biomarkers of infection. Furthermore, the characterization and confirmation of critical protein–protein interaction residues by integrating computational approaches, such as MD simulations, with experimental validation can facilitate the development of antibody-based detection methods or biosensor platforms for *T. marneffei*.

In summary, this work enhances our understanding of the molecular mechanisms underlying host–pathogen interactions and opens new avenues for future studies to unravel how fungal pathogens subvert host cell architecture and trafficking pathways.

## Figures and Tables

**Table 1:** The TmGpx1 protein interactome was identified by yeast two-hybrid assay.

PBS	# of Positive Clones	Gene Name	Function
A	45/258	ACTN1	Actin-associated protein
	13/258	BRAP	BRCA1-associated protein, regulating nuclear targeting by retaining proteins with a nuclear localization signal in the cytoplasm
	31/258	CSNK2B	Casein kinase 2 Beta
	14/258	FKBP15	Protein involved in cytoskeleton organization, and transition between microfilament-based and microtubule-based movement.
	8/258	MLX	Transcription factor, Max dimerization protein, plays a role in proliferation, determination, and differentiation.
	11/258	MOB1A	MOB kinase activator 1A, involved in the Hippo signaling pathway and the control of microtubule stability during cytokinesis.
	20/258	NUMA1	Protein interacting with microtubule, involved in the formation and organization of the mitotic spindle during cell division.
	20/258	TMEM109	Transmembrane protein
B	5/258	DYNC1H1	Heavy chain of cytoplasmic dynein-1
	6/258	TRIO	GDP-to-GTP exchange factor that promotes the reorganization of the actin skeleton during cell growth and migration.
	5/258	TXNL1	Thioredoxin-like protein, having dual functions in disulfide reduction activity and chaperone properties.
C	3/258	CLIP2	Cytoplasmic linker protein, associated with microtubules and membranous organelles.
	4/258	DNAJB11	Heat shock protein, a co-chaperone of Ig protein/ER processing.
	3/258	MYH9	Myosin heavy chain 9, subunit of myosin IIA

**Table 2:** Gene ontology enrichment analysis of Gpx1 protein interacting partners. Sixteen Gpx1 protein partners were analyzed using the PANTHER (Protein ANalysis THrough Evolutionary Relationships) Classification System (https://geneontology.org/ and https://pantherdb.org/, accessed on 31 January 2026).

GO Cellular Component	# Human	# Y2H	Gene	Fold Enrichment
Microtubule plus-end	26	2	CLIP2 NUMA1	98.94
Cortical cytoskeleton	108	3	NUMA1 ACTN1 MYH9	35.73
Cell cortex	324	5	CLIP2 DYNC1H1 NUMA1 ACTN1 MYH9	19.85
Extracellular exosome - Extracellular vesicle - Extracellular membrane-bounded organelle - Extracellular organelle	2102	8	CSNK2B DYNC1H1 NUMA1 ACTN1 MOB1A/B MYH9 TMEM109	4.9

**Table 3:** MM-GBSA (GB model) binding free energy components (mean ± SD, kcal/mol) calculated from GMX_MMPBSA Δ (complex − TmGpx1 − Fkbp15) over frames 90–100. Calculation was performed in triplicate and reported as mean ± standard deviation.

Component	Mean ± SD (kcal/mol)
**Gas phase (GGAS)**	
ΔE_vdw (VDWAALS)	−182.28 ± 41.29
ΔE_elect (EEL)	−744.01 ± 300.03
**Solvent phase (GSOLV)**	
ΔG_polar (EGB)	864.63 ± 304.76
ΔG_nonpolar (ESURF)	−25.93 ± 5.93
**ΔG_bind (TOTAL) = GGAS + GSOLV**	**−** **87.58 ± 32.43**

## Data Availability

All supplemental data used to generate tables and figures in this manuscript are available in the Supplementary Materials.

## Supplementary Materials

The following supporting information can be downloaded at: https://www.mdpi.com/article/10.3390/ijms27104259/s1.
