# Supplementary material for: Glutathione Peroxidase from Talaromyces marneffei Interacts with Host Cytoskeletal Proteins: Insights from Yeast Two-Hybrid and Molecular Dynamics Simulations
Source: Int J Mol Sci. 2026 May 11;27(10):4259. doi: 10.3390/ijms27104259 (PMC13207274; doi:10.3390/ijms27104259)

HYBRIGENICS SERVICES

DomSight: MAC_RP1_hgx6974v1 vs. Macrophage_RP1 (11 Dec 2024)

(Bait plasmid(s): hgx6974v1_pB27)

Bait fragment
SID fragment
Pfam or SMART domain
Transmembrane domain (TMHMM, Phobius)
Coiled-coil domain (Ncoils)
Signal peptide (SignalP, Phobius)
Cytoplasmic domain (Phobius)
Non cytoplasmic domain (Phobius)

SID: Selected Interaction Domain
It is the amino acid sequence shared by all prey fragments matching the same reference protein. SIDs have been found in numerous cases to correspond to an identified structural or functional domain.
GenMatch/NoMatch SIDs are shortened at the first in-frame stop codon.
Protein Display:
Proteins larger than 1500 aa are shown in sections of 1499 aa each.
Only sections containing bait fragments, SIDs or predicted functional and structural domains are represented.

Legend

glutathione peroxidase Hyr1, putative — Talaromyces marneffei ATCC 18224 (1–197)
- 1 197 hgx6974v1
- 7 117 Glutathione peroxidase - PF00255

D ACLY — Homo sapiens, ACCESSION: NM_001096.3, Gene ID: 47 (1–1101)
- 119 315 SID
- 244 420 ATP-citrate synthase, citrate-binding do - PF16114
- 492 601 CoA-binding - SM00881
- 495 600 CoA-binding - PF02629
- 660 784 ATP-citrate synthase/succinyl-CoA ligase - PF00549
- 888 1081 Citrate synthase - PF00285

A ACTN1 var1 — Homo sapiens, ACCESSION: NM_001130004.2, Gene ID: 87 (1–914)
- 371 586 SID
- 265 292 Coiled domain
- 430 464 Coiled domain
- 32 135 Calponin homology domain - PF00307
- 33 133 Calponin homology domain - SM00033
- 145 250 Calponin homology domain - PF00307
- 146 245 Calponin homology domain - SM00033
- 275 383 Spectrin repeat - PF00435
- 394 498 Spectrin repeat - PF00435
- 397 498 Spectrin/alpha-actinin - SM00150
- 510 620 Spectrin repeat - PF00435
- 512 619 Spectrin/alpha-actinin - SM00150
- 632 732 Spectrin repeat - PF00435
- 633 732 Spectrin/alpha-actinin - SM00150
- 750 778 EF-hand domain - SM00054
- 750 779 EF-hand domain - PF13405
- 790 840 EF-hand domain - PF13833
- 813 841 EF-hand domain - SM00054
- 844 910 efhand_Ca_insen_2 - SM01184
- 844 910 EF-hand, Ca insensitive - PF08726

D BAG4 — Homo sapiens, ACCESSION: NM_004874.4, Gene ID: 9530 (1–457)
- 139 444 SID
- 379 456 BAG domain - SM00264
- 385 455 BAG domain - PF02179

A BRAP — Homo sapiens, ACCESSION: NM_006768.5, Gene ID: 8315 (1–592)
- 476 592 SID

C CLIP2 — Homo sapiens, ACCESSION: NM_032421.3, Gene ID: 7461 (1–1011)
- 357 430 SID
- 354 381 Coiled domain
- 396 455 Coiled domain
- 529 577 Coiled domain
- 640 667 Coiled domain
- 669 765 Coiled domain
- 818 852 Coiled domain
- 901 928 Coiled domain
- 948 975 Coiled domain
- 81 146 CAP Gly-rich domain - SM01052
- 81 145 CAP Gly-rich domain - PF01302
- 221 285 CAP Gly-rich domain - PF01302
- 221 286 CAP Gly-rich domain - SM01052

A CSNK2B — Homo sapiens, ACCESSION: NM_001320.7, Gene ID: 1460 (1–215)
- 1 162 SID
- 8 191 Casein kinase II, regulatory subunit - PF01214
- 8 191 Casein kinase II, regulatory subunit - SM01085

D CTSB — Homo sapiens, ACCESSION: NM_001908.5, Gene ID: 1508 (1–339)
- 120 301 SID
- 1 19 Signal peptide domain (phobius)
- 1 17 Signal peptide domain (signalp)
- 20 339 Non Cytoplasmic domain (phobius)
- 26 63 Peptidase C1A, propeptide - PF08127
- 80 329 Peptidase C1A, papain C-terminal - SM00645
- 80 328 Peptidase C1A, papain C-terminal - PF00112

C DNAJB11 — Homo sapiens, ACCESSION: NM_016306.6, Gene ID: 51726 (1–358)
- 117 331 SID
- 5 27 Transmembrane domain (tmhmm)
- 1 22 Signal peptide domain (phobius)
- 1 22 Signal peptide domain (signalp)
- 23 358 Non Cytoplasmic domain (phobius)
- 24 82 DnaJ domain - SM00271
- 25 87 DnaJ domain - PF00226
- 136 327 Chaperone DnaJ, C-terminal - PF01556

B DYNC1H1 — Homo sapiens, ACCESSION: NM_001376.5, Gene ID: 1778 (1500–2999; 3000–4499; 4500–4646)
- 2032 2193 SID

A FKBP15 — Homo sapiens, ACCESSION: NM_015258.2, Gene ID: 23307 (1–1219)
- 506 695 SID
- 567 608 Coiled domain
- 624 658 Coiled domain
- 687 785 Coiled domain
- 824 872 Coiled domain
- 926 951 Coiled domain
- 194 286 FKBP-type peptidyl-prolyl cis-trans isom - PF00254
- 556 779 FK506-binding protein 15-like domain - PF23649

D HELZ — Homo sapiens, ACCESSION: NM_014877.4, Gene ID: 9931 (1–1499; 1500–1942)
- 1388 1499 SID
- 178 205 Zinc finger, CCCH-type - SM00356
- 181 205 Zinc finger, CCCH-type - PF00642
- 643 754 UvrD-like helicase, ATP-binding domain - PF00580
- 759 833 DNA2/NAM7 helicase, helicase domain - PF13086
- 841 1050 DNA2/NAM7 helicase-like, C-terminal - PF13087
- 1500 1534 SID

E IQGAP1 — Homo sapiens, ACCESSION: NM_003870.4, Gene ID: 8826 (1–1499; 1500–1657)
- 329 697 SID
- 168 195 Coiled domain
- 263 297 Coiled domain
- 368 388 Coiled domain
- 487 514 Coiled domain
- 889 909 Coiled domain
- 1496 1499 Coiled domain
- 46 155 Calponin homology domain - SM00033
- 48 158 Calponin homology domain - PF00307
- 680 712 WW domain - SM00456
- 744 766 IQ motif, EF-hand binding site - SM00015
- 749 766 IQ motif, EF-hand binding site - PF00612
- 774 796 IQ motif, EF-hand binding site - SM00015
- 778 796 IQ motif, EF-hand binding site - PF00612
- 804 826 IQ motif, EF-hand binding site - SM00015
- 809 826 IQ motif, EF-hand binding site - PF00612
- 834 856 IQ motif, EF-hand binding site - SM00015
- 839 856 IQ motif, EF-hand binding site - PF00612
- 992 1345 Ras GTPase-activating domain - SM00323
- 1025 1237 Ras GTPase-activating domain - PF00616
- 1452 1499 RasGAP protein, C-terminal - PF03836
- 1500 1516 Coiled domain
- 1500 1580 RasGAP protein, C-terminal - PF03836

HYBRIGENICS SERVICES

DomSight: MAC_RP1_hgx6974v1 vs. Macrophage_RP1 (11 Dec 2024)

(Bait plasmid(s): hgx6974v1_pB27)

Bait fragment
SID fragment
Pfam or SMART domain
Transmembrane domain (TMHMM, Phobius)
Coiled-coil domain (Ncoils)
Signal peptide (SignalP, Phobius)
Cytoplasmic domain (Phobius)
Non cytoplasmic domain (Phobius)

SID: Selected Interaction Domain
It is the amino acid sequence shared by all prey fragments matching the same reference protein. SIDs have been found in numerous cases to correspond to an identified structural or functional domain.
GenMatch/NoMatch SIDs are shortened at the first in-frame stop codon.
Protein Display:
Proteins larger than 1500 aa are shown in sections of 1499 aa each.
Only sections containing bait fragments, SIDs or predicted functional and structural domains are represented.

Legend

D KDELR1
Homo sapiens
ACCESSION: NM_006801.3
Gene ID: 10945

1 212
1 6 SID
6 24 Transmembrane domain (phobius)
36 59 Transmembrane domain (phobius)
56 78 Transmembrane domain (tmhmm)
65 82 Transmembrane domain (phobius)
94 113 Transmembrane domain (phobius)
116 138 Transmembrane domain (tmhmm)
119 138 Transmembrane domain (phobius)
150 168 Transmembrane domain (phobius)
151 168 Transmembrane domain (tmhmm)
174 199 Transmembrane domain (phobius)
178 200 Transmembrane domain (tmhmm)
25 35 Cytoplasmic domain (phobius)
83 93 Cytoplasmic domain (phobius)
139 149 Cytoplasmic domain (phobius)
200 212 Cytoplasmic domain (phobius)
1 5 Non Cytoplasmic domain (phobius)
60 64 Non Cytoplasmic domain (phobius)
114 118 Non Cytoplasmic domain (phobius)
169 173 Non Cytoplasmic domain (phobius)
28 169 ER lumen protein retaining receptor - PF00810

D KLC2
Homo sapiens
ACCESSION: NM_001134775.2
Gene ID: 64837

1 622
1 622 SID
85 140 Coiled domain
198 272 Tetratricopeptide repeat - PF13424
240 273 Tetratricopeptide repeat - SM00028
281 354 Tetratricopeptide repeat - PF13424
282 315 Tetratricopeptide repeat - SM00028
324 357 Tetratricopeptide repeat - SM00028
365 402 Tetratricopeptide repeat - PF13374
366 399 Tetratricopeptide repeat - SM00028

E MACF1
Homo sapiens
ACCESSION: NP_036222.3
Gene ID: 23499

1 1499
245 265 Coiled domain
477 497 Coiled domain
502 529 Coiled domain
821 841 Coiled domain
1046 1066 Coiled domain
1403 1433 Coiled domain
1497 1499 Coiled domain
79 181 Calponin homology domain - PF00307
80 179 Calponin homology domain - SM00033
195 298 Calponin homology domain - PF00307
196 293 Calponin homology domain - SM00033
425 522 Plectin-like, spectrin-like repeat - PF21020
583 680 Spectrin/alpha-actinin - SM00150
683 783 Spectrin/alpha-actinin - SM00150
789 856 Spectrin-like repeat - PF21019
857 923 Desmoplakin, SH3 domain - PF17902
958 1035 Desmoplakin, spectrin-like domain - PF18373
1042 1161 Spectrin repeat - PF21097
1278 1407 Spectrin/alpha-actinin - SM00150
1425 1499 Spectrin/alpha-actinin - SM00150

1500 2999
1500 1517 Coiled domain
1522 1549 Coiled domain
1629 1663 Coiled domain
1793 1823 Coiled domain
2187 2214 Coiled domain
2234 2254 Coiled domain
2292 2312 Coiled domain
2397 2417 Coiled domain
2557 2577 Coiled domain
2616 2636 Coiled domain
2657 2691 Coiled domain
2808 2828 Coiled domain
2929 2970 Coiled domain
1500 1533 Spectrin/alpha-actinin - SM00150
1550 1658 Spectrin/alpha-actinin - SM00150
1816 1890 Spectrin repeat - PF00435
1818 1928 Spectrin/alpha-actinin - SM00150
1933 2041 Spectrin repeat - PF00435
1935 2041 Spectrin/alpha-actinin - SM00150
2048 2150 Spectrin/alpha-actinin - SM00150
2157 2256 Spectrin/alpha-actinin - SM00150
2263 2394 Spectrin/alpha-actinin - SM00150
2363 2431 DELLA_2 - SM01129
2399 2507 Spectrin repeat - PF00435
2401 2506 Spectrin/alpha-actinin - SM00150
2513 2617 Spectrin/alpha-actinin - SM00150
2624 2727 Spectrin/alpha-actinin - SM00150
2733 2837 Spectrin repeat - PF00435
2734 2837 Spectrin/alpha-actinin - SM00150
2842 2945 Spectrin repeat - PF00435
2844 2944 Spectrin/alpha-actinin - SM00150

3000 4499
4258 4499 SID
3133 3153 Coiled domain
3244 3264 Coiled domain
3897 3917 Coiled domain
4133 4160 Coiled domain
4345 4365 Coiled domain
3060 3162 Spectrin/alpha-actinin - SM00150
3169 3273 Spectrin/alpha-actinin - SM00150
3169 3274 Spectrin repeat - PF00435
3280 3382 Spectrin/alpha-actinin - SM00150
3280 3383 Spectrin repeat - PF00435
3388 3491 Spectrin repeat - PF00435
3389 3491 Spectrin/alpha-actinin - SM00150
3498 3600 Spectrin/alpha-actinin - SM00150
3607 3709 Spectrin/alpha-actinin - SM00150
3716 3818 Spectrin/alpha-actinin - SM00150
3825 3926 Spectrin/alpha-actinin - SM00150
3826 3927 Spectrin repeat - PF00435
3936 4042 Spectrin/alpha-actinin - SM00150
4047 4152 Spectrin repeat - PF00435
4049 4151 Spectrin/alpha-actinin - SM00150
4157 4261 Spectrin repeat - PF00435
4158 4261 Spectrin/alpha-actinin - SM00150
4267 4370 Spectrin repeat - PF00435
4268 4370 Spectrin/alpha-actinin - SM00150
4375 4481 Spectrin repeat - PF00435
4377 4480 Spectrin/alpha-actinin - SM00150
4485 4499 Spectrin repeat - PF00435
4487 4499 Spectrin/alpha-actinin - SM00150

4500 5430
4500 5430 SID
4912 4932 Coiled domain
4500 4589 Spectrin repeat - PF00435
4500 4589 Spectrin/alpha-actinin - SM00150
4594 4700 Spectrin repeat - PF00435
4596 4699 Spectrin/alpha-actinin - SM00150
4706 4807 Spectrin/alpha-actinin - SM00150
4707 4808 Spectrin repeat - PF00435
4813 4916 Spectrin repeat - PF00435
4814 4916 Spectrin/alpha-actinin - SM00150
4923 5053 Spectrin/alpha-actinin - SM00150
5086 5147 EF-hand domain - PF13499
5087 5115 EF-hand domain - SM00054
5123 5151 EF-hand domain - SM00054
5162 5234 GAR domain - SM00243
5164 5232 GAR domain - PF02187

A MLX
Homo sapiens
ACCESSION: NM_198204.2
Gene ID: 6945

1 244
46 244 SID
69 89 Coiled domain
130 157 Coiled domain
76 133 Myc-type, basic helix-loop-helix (bHLH) - PF00010
81 139 Myc-type, basic helix-loop-helix (bHLH) - SM00353

D MMP9
Homo sapiens
ACCESSION: NM_004994.3
Gene ID: 4318

1 707
173 243 SID
1 19 Signal peptide domain (phobius)
1 19 Signal peptide domain (signalp)
20 707 Non Cytoplasmic domain (phobius)
112 445 Peptidase, metallopeptidase - SM00235
115 214 Peptidase M10, metallopeptidase - PF00413
223 271 Fibronectin type II domain - SM00059
230 271 Fibronectin type II domain - PF00040
281 329 Fibronectin type II domain - SM00059
288 329 Fibronectin type II domain - PF00040
340 388 Fibronectin type II domain - SM00059
347 388 Fibronectin type II domain - PF00040
389 444 Peptidase M10, metallopeptidase - PF00413
521 565 Hemopexin-like repeats - SM00120
521 564 Hemopexin-like repeats - PF00045
567 608 Hemopexin-like repeats - SM00120
568 606 Hemopexin-like repeats - PF00045
613 659 Hemopexin-like repeats - SM00120
615 655 Hemopexin-like repeats - PF00045
661 704 Hemopexin-like repeats - SM00120
663 704 Hemopexin-like repeats - PF00045

DomSight: MAC_RP1_hgx6974v1 vs. Macrophage_RP1 (11 Dec 2024)

(Bait plasmid(s): hgx6974v1_pB27)

Bait fragment
SID fragment
Pfam or SMART domain
Transmembrane domain (TMHMM, Phobius)
Coiled-coil domain (Ncoils)
Signal peptide (SignalP, Phobius)
Cytoplasmic domain (Phobius)
Non cytoplasmic domain (Phobius)

SID: Selected Interaction Domain
It is the amino acid sequence shared by all prey fragments matching the same reference protein. SIDs have been found in numerous cases to correspond to an identified structural or functional domain.
GenMatch/NoMatch SIDs are shortened at the first in-frame stop codon.
Protein Display:
Proteins larger than 1500 aa are shown in sections of 1499 aa each.
Only sections containing bait fragments, SIDs or predicted functional and structural domains are represented.

Legend

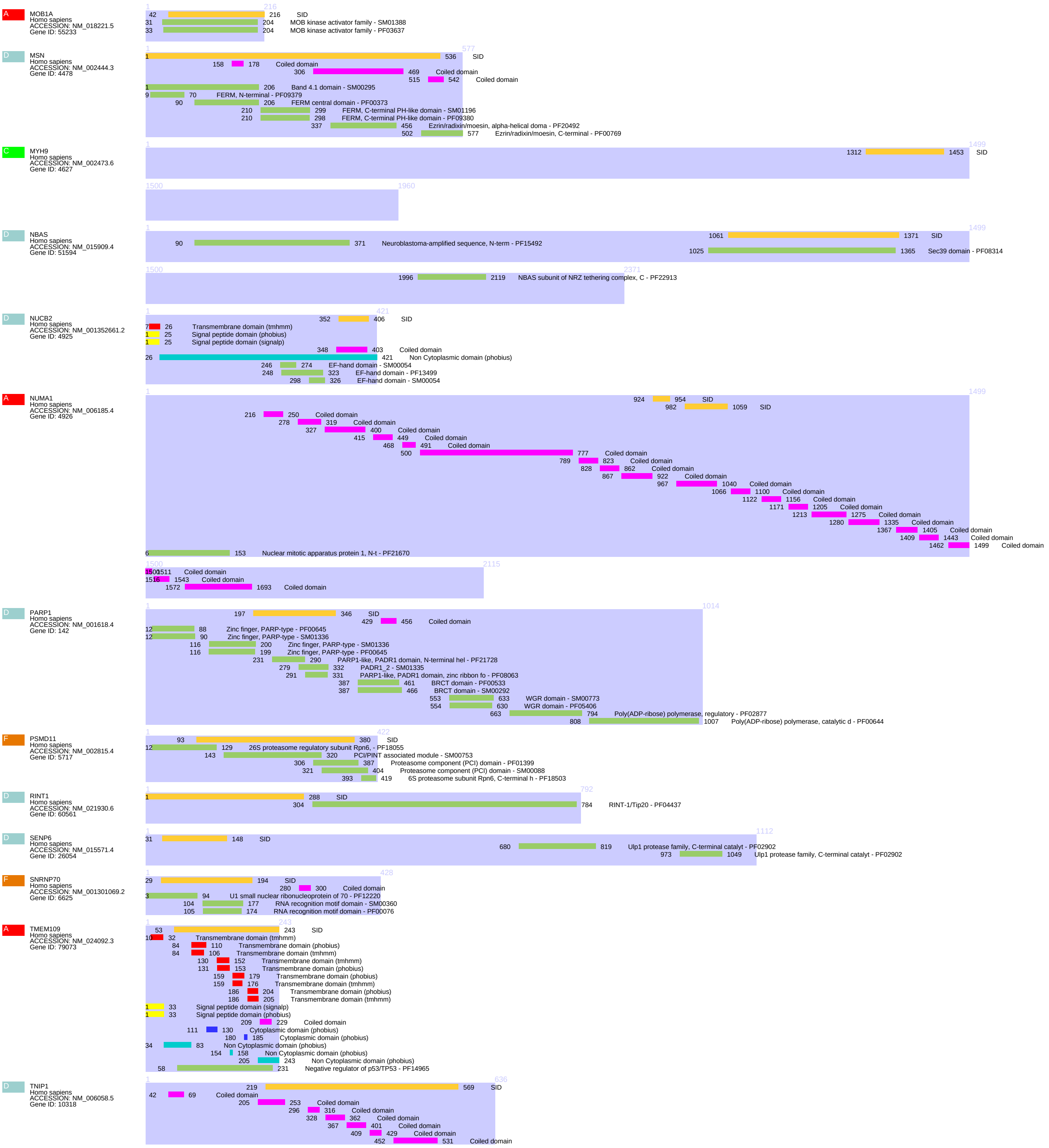

DomSight: MAC_RP1_hgx6974v1 vs. Macrophage_RP1 (11 Dec 2024)

(Bait plasmid(s): hgx6974v1_pB27)

Bait fragment
SID fragment
Pfam or SMART domain
Transmembrane domain (TMHMM, Phobius)
Coiled-coil domain (Ncoils)
Signal peptide (SignalP, Phobius)
Cytoplasmic domain (Phobius)
Non cytoplasmic domain (Phobius)

SID: Selected Interaction Domain
It is the amino acid sequence shared by all prey fragments matching the same reference protein. SIDs have been found in numerous cases to correspond to an identified structural or functional domain.
GenMatch/NoMatch SIDs are shortened at the first in-frame stop codon.
Protein Display:
Proteins larger than 1500 aa are shown in sections of 1499 aa each. Only sections containing bait fragments, SIDs or predicted functional and structural domains are represented.

Legend

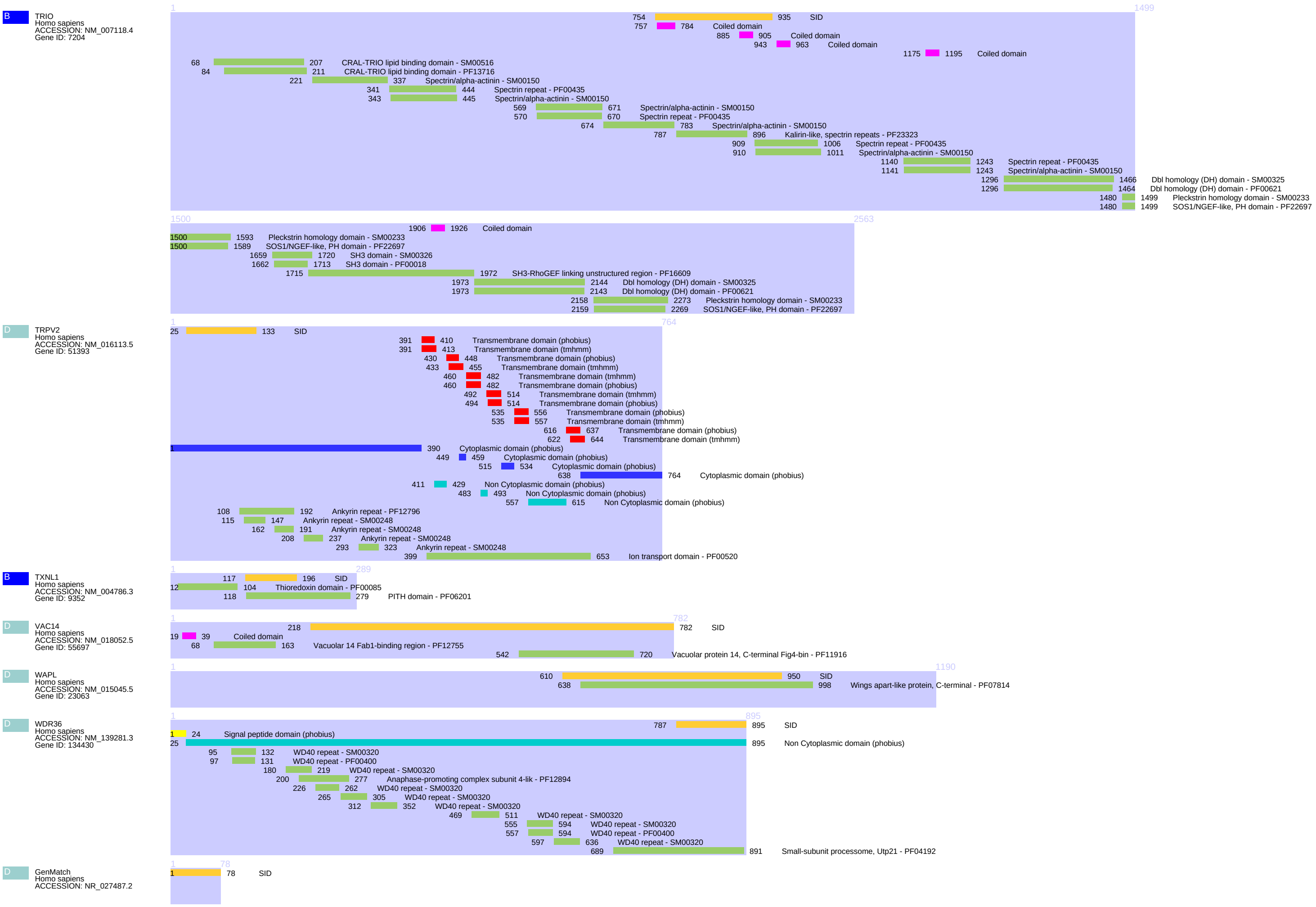

Supplement: Supplementary file 1 [file ijms-27-04259-s001.zip › ijms-4249107-supplementary/Supplemental Data S1.pdf]
