# Supplementary material for: Glutathione Peroxidase from Talaromyces marneffei Interacts with Host Cytoskeletal Proteins: Insights from Yeast Two-Hybrid and Molecular Dynamics Simulations
Source: Int J Mol Sci. 2026 May 11;27(10):4259. doi: 10.3390/ijms27104259 (PMC13207274; doi:10.3390/ijms27104259)
Supplement: Supplementary file 1 [file ijms-27-04259-s001.zip › ijms-4249107-supplementary/Supplemental Data S2.pdf]

Gpx (197 aa)
>EEA19957.1 glutathione peroxidase Hyr1, putative [*Talaromyces marneffei* ATCC 18224]
MASATTFYDFSPPDKKGNPYPLTDYK**GKVVLVVNTASKCGFT**PQFAGLEKLYKSIEAKHPGAFTILGFPCNQFGNQD
PGSNDEIQSFCQVNYGVTFPVLGKIDVNGSKAEPLFEWIKSEKPGLLGVKRVLWNFEKALINGKGEVVGRWRSITKP
ESLEATILKEIDIASKDVKGVEVVPTATETAASAAPAEEAKEA

FKBP15 (1219 aa)
>NP_056073.1 FK506-binding protein 15 [*Homo sapiens*]
MFGAGDEDDTDFLSPSGGARLASLFGLDQAAAGHGNEFFQYTAPKQPKKGQGTAATGNQATPKTAPATMSTPTILVA
TAVHAYRYTNGQYVKQGKFGAAVLGNHTAREYRILLYISQQQPVTVARIHVNFELMVRPNNYSTFYDDQRQNWSIMF
ESEKAAVEFNKQVCIAKCNSTSSLDAVLSQDLIVADGPAVEVGDSLEVAYTGWLFQNHVLGQVFDSTANKDKLLRLK
LGSGKVIKGWEDGMLGMKKGGKRLLIVPPACAVGSEGVIGWTQATDSILVFEVEVRRVKFARDSGSDGHSVSSRDSA
APSPIPGADNLSADPVVSPPTSIPFKSGEPALRTKSNSLSEQLAINTSPDAVKAKLISRMAKMGQPMLPILPPQLDS
NDSEIEDVNTLQGGGQPVVTPSVQPSLHPAHPALPQMTSQAPQPSVTGLQAPSAALMQVSSLDSHSAVSGNAQSFQP
YAGMQAYAYPQASAVTSQLQPVRPLYPAPLSQPPHFQGSGDMASFLMTEARQHNTEIRMAVSKVADKMDHLMTKVEE
LQKHSAGNSMLIPSMSVTMETSMIMSNIQRIIQENERLKQEILEKSNRIEEQNDKISELIERNQRYVEQSNLMMEKR
NNSLQTATENTQARVLHAEQEKAKVTEELAAATAQVSHLQLKMTAHQKKETELQMQLTESLKETDLLRGQLTKVQAK
LSELQETSEQAQSKFKSEKQNRKQLELKVTSLEEELTDLRVEKESLEKNLSERKKKSAQERSQAEEEIDEIRKSYQE
ELDKLRQLLKKTRVSTDQAAAEQLSLVQAELQTQWEAKCEHLLASAKDEHLQQYQEVCAQRDAYQQKLVQLQEKCLA
LQAQITALTKQNEQHIKELEKNKSQMSGVEAAASDPSEKVKKIMNQVFQSLRREFELEESYNGRTILGTIMNTIKMV
TLQLLNQQEQEKEESSSEEEEEKAEERPRRPSQEQSASASSGQPQAPLNRERPESPMVPSEQVVEEAVPLPPQALTT
SQDGHRRKGDSEAEALSEIKDGSLPPELSCIPSHRVLGPPTSIPPEPLGPVSMDSECEESLAASPMAAKPDNPSGKV
CVREVAPDGPLQESSTRLSLTSDPEEGDPLALGPESPGEPQPPQLKKDDVTSSTGPHKELSSTEAGSTVAGAALRPS
HHSQRSSLSGDEEDELFKGATLKALRPKAQPEEEDEDEVSMKGRPPPTPLFGDDDDDDDIDWLG

Label
Bold: Gpx active site
Yellow highlight: Gpx catalytic triad
Green highlight: Gpx protein sequences used in MD simulation
Blue highlight: FKBP15 protein sequences used in MD simulation
