# Supplementary material for: Glutathione Peroxidase from Talaromyces marneffei Interacts with Host Cytoskeletal Proteins: Insights from Yeast Two-Hybrid and Molecular Dynamics Simulations
Source: Int J Mol Sci. 2026 May 11;27(10):4259. doi: 10.3390/ijms27104259 (PMC13207274; doi:10.3390/ijms27104259)
Supplement: Supplementary file 1 [file ijms-27-04259-s001.zip › ijms-4249107-supplementary/Supplemental Figures.pdf]

Glutathione Peroxidase from *Talaromyces marneffei* Interacts with Host Cytoskeletal Proteins: Insights from Yeast Two-Hybrid and Molecular Dynamics Simulation

Tanaporn Wangsanut[1], Yin Htet Htet Aung[1], Yin Htet Htet Oo[1], Narin Lawan[2], Monsicha Pongpom[1]*

[1] Department of Microbiology, Faculty of Medicine, Chiang Mai University, Chiang Mai, Thailand 50200

[2] Department of Chemistry, Faculty of Sciences, Chiang Mai University, Chiang Mai, Thailand 50200

* Correspondence: monsicha.p@cmu.ac.th

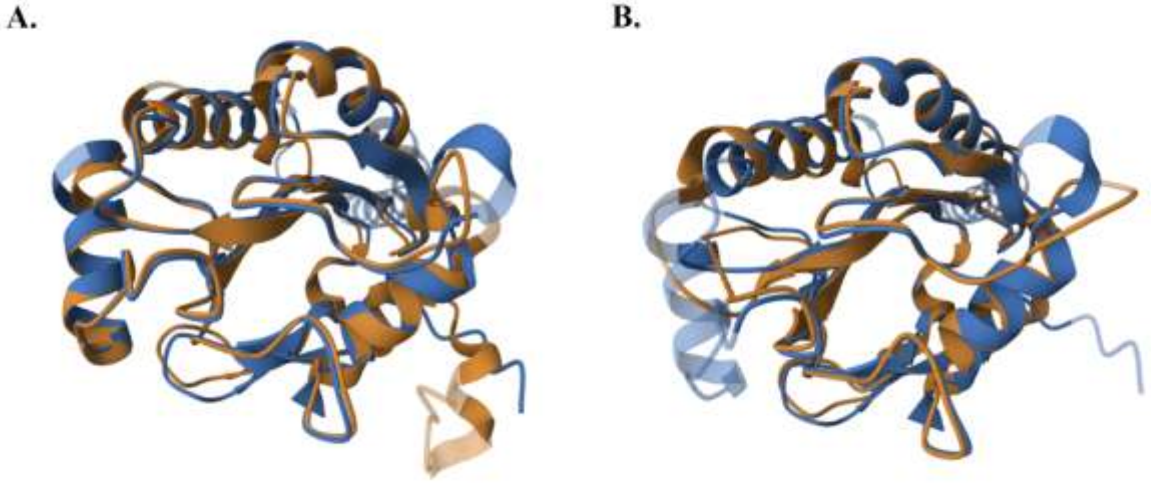

Figure S1. Structural superimposition of predicted TmGpx1 with experimentally determined homologs. Superimposition of the AlphaFold3-predicted TmGpx1 structure onto (A) human GPx4 (PDB: 2GS3, chain A), and (B) yeast Hyr1 (PDB: 3CMI, chain A) was performed using the RCSB PDB structure-alignment tool. The alignments yielded RMSD/TM-score/sequence identity (aligned residues) of 1.11 Å/0.90/38% (160 residues) for 2GS3 and 1.67 Å/0.92/54% (140 residues) for 3CMI (Table S1). Blue represents TmGpx1, Brown represents Human GPx4 in A. or yeast Hyr1 in B.

A.

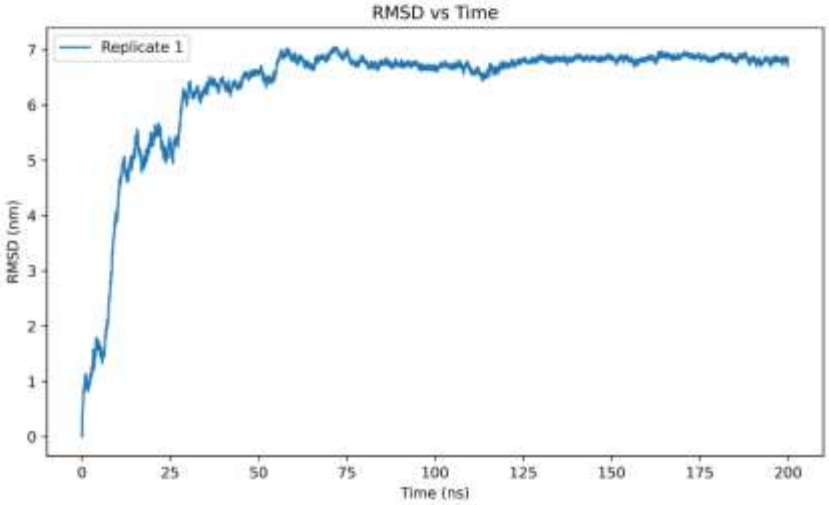

RMSD vs Time
Replicate 1
RMSD (nm)
0
1
2
3
4
5
6
7
Time (ns)
0
25
50
75
100
125
150
175
200

B.

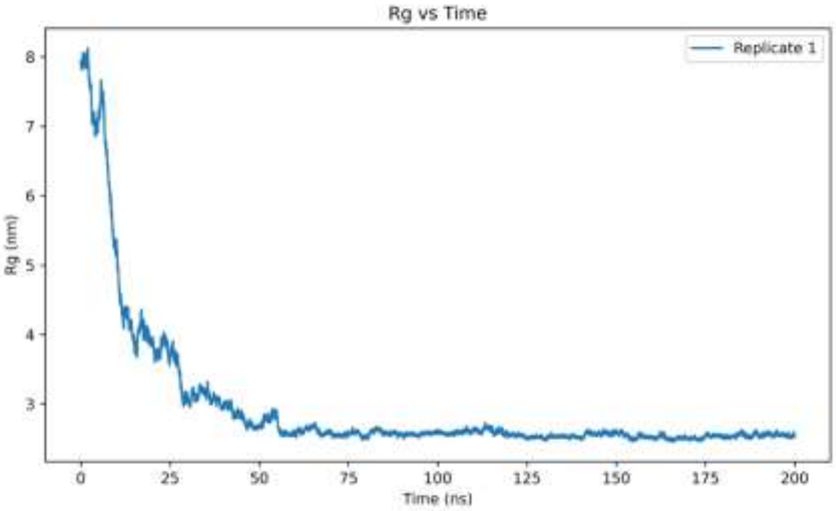

Rg vs Time
Replicate 1
Rg (nm)
3
4
5
6
7
8
Time (ns)
0
25
50
75
100
125
150
175
200

C.

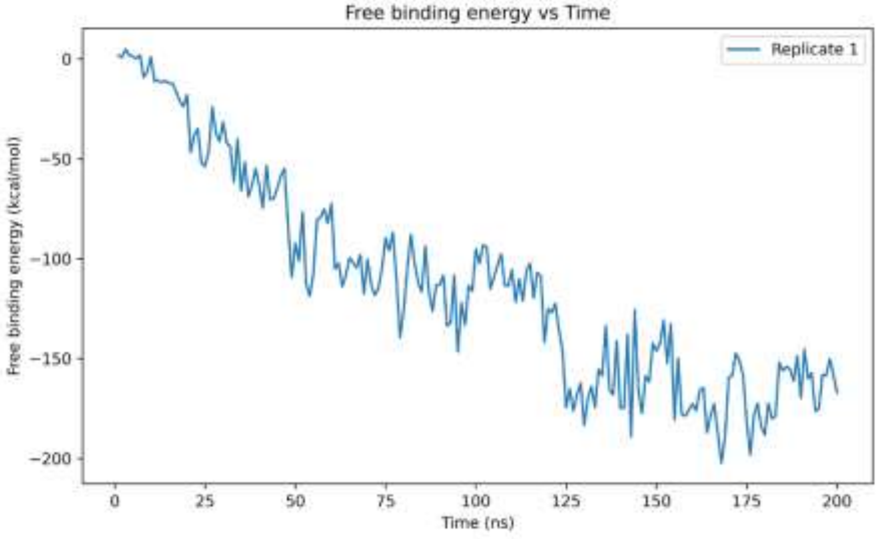

Free binding energy vs Time
Replicate 1
Free binding energy (kcal/mol)
0
−50
−100
−150
−200
Time (ns)
0
25
50
75
100
125
150
175
200

Figure S2. Convergence assessment using an extended 200 ns MD trajectory. To evaluate whether 100 ns per replica is sufficient for this protein–protein complex, replicate 1 was extended to 200 ns and structural stability metrics were monitored over time. (A) Protein RMSD (nm) of the TmGpx1–FKBP15 complex over the 200 ns trajectory. (B) Radius of gyration (Rg, nm) of the complex over the 200 ns trajectory. Both metrics indicate that the system reaches a stable regime by ~100 ns with no further systematic drift thereafter. (C) MM/PBSA binding free energy (kcal/mol) calculated along the full 200 ns trajectory; the average binding free energy over 200 ns is −112.42 kcal/mol.

A.

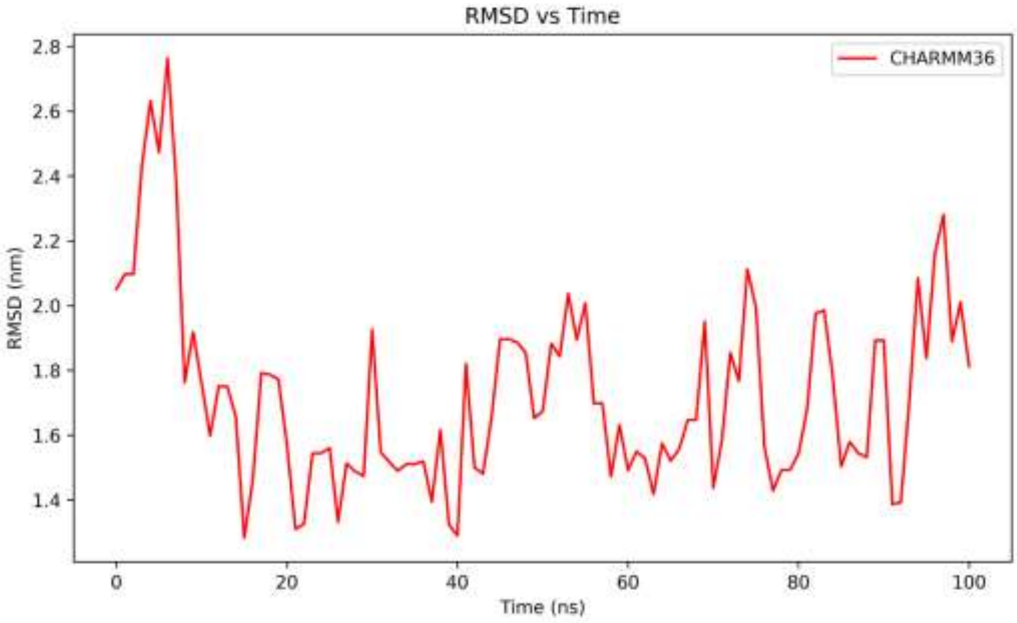

B.

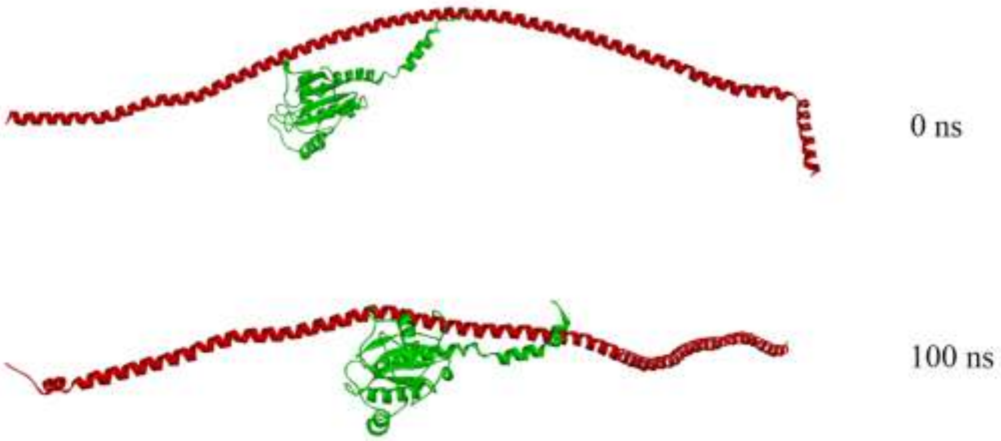

Figure S3. CHARMM36m simulation of the TmGpx1–FKBP15 protein complex. (A) Protein RMSD (nm) of the TmGpx1–FKBP15 complex during the 100 ns MD simulation performed with the CHARMM36m force field, calculated relative to the starting structure (0 ns). (B) Representative 3D structures (snapshots) of the TmGpx1–FKBP15 complex at 0 ns and 100 ns from the CHARMM36m trajectory.
