# Supplementary material for: Glutathione Peroxidase from Talaromyces marneffei Interacts with Host Cytoskeletal Proteins: Insights from Yeast Two-Hybrid and Molecular Dynamics Simulations
Source: Int J Mol Sci. 2026 May 11;27(10):4259. doi: 10.3390/ijms27104259 (PMC13207274; doi:10.3390/ijms27104259)
Supplement: Supplementary file 1 [file ijms-27-04259-s001.zip › ijms-4249107-supplementary/Supplemental Tables.pdf]

Table S1. Structural similarity of predicted TmGpx1 to experimental homologs (RCSB alignment).

Experimental homolog (PDB)	Chain	RMSD (Å)	TM-score	Seq. identity	Aligned residues
Human GPx4 (2GS3)	A	1.11	0.90	38%	160
Yeast Hyr1 (3CMI)	A	1.67	0.92	54%	140

Table S2. MM-GBSA (GB model) binding free energy components (mean ± SD, kcal/mol) calculated from GMX_MMPBSA Δ (complex – TmGpx1 – Fkbp15) over frames 90–100. Calculation was performed in three replicates.

**Component**	**Mean ± SD (kcal/mol)**
Replicate 1	
**Gas phase (GGAS)**	
ΔE_vdw (VDWAALS)	−222.47 ± 14.68
ΔE_elect (EEL)	−1008.24 ± 86.75
**Solvent phase (GSOLV)**	
ΔG_polar (EGB)	1143.07 ± 82.74
ΔG_nonpolar (ESURF)	−32.60 ± 2.39
**ΔG_bind (TOTAL) = GGAS + GSOLV**	**−120.24 ± 14.18**
Replicate 2	
**Gas phase (GGAS)**	
ΔE_vdw (VDWAALS)	-139.98 ± 8.39
ΔE_elect (EEL)	-805.93 ± 48.90
**Solvent phase (GSOLV)**	
ΔG_polar (EGB)	911.77 ± 40.39
ΔG_nonpolar (ESURF)	-21.25 ± 1.05
**ΔG_bind (TOTAL) = GGAS + GSOLV**	**-55.39 ± 8.91**
Replicate 3	
**Gas phase (GGAS)**	
ΔE_vdw (VDWAALS)	-184.38 ± 7.04
ΔE_elect (EEL)	-417.85 ± 28.69
**Solvent phase (GSOLV)**	
ΔG_polar (EGB)	539.04 ± 25.51
ΔG_nonpolar (ESURF)	-23.93 ± 0.74
**ΔG_bind (TOTAL) = GGAS + GSOLV**	**-87.12 ± 8.09**
